# Needs, Requirements and a Concept of a Tool Condition Monitoring System for the Aerospace Industry

**DOI:** 10.3390/s21155086

**Published:** 2021-07-27

**Authors:** Sebastian Bombiński, Joanna Kossakowska, Mirosław Nejman, Rodolfo E. Haber, Fernando Castaño, Robert Fularski

**Affiliations:** 1 Faculty of Mechanical Engineering, Kazimierz Pulaski University of Technology and Humanities in Radom, 26-610 Radom, Poland; 2 Dept. of Automation and Metal Cutting, Warsaw University of Technology, 02-524 Warsaw, Poland; j.kossakowska@pw.edu.pl (J.K.); miroslaw.nejman@pw.edu.pl (M.N.); 3 Centre for Automation and Robotics, Spanish National Research Council-Technical University of Madrid, 28500 Madrid, Spain; rodolfo.haber@car.upm-csic.es (R.E.H.); fernando.castano@car.upm-csic.es (F.C.); 4 Pratt & Whitney Rzeszów, 35-001 Rzeszów, Poland; robert.fularski@prattwhitney.com

**Keywords:** tool condition monitoring system (TCM), tool wear, supervision system, cyber-physical system (CPS), digital twin

## Abstract

In this paper, we describe the needs and specific requirements of the aerospace industry in the field of metal machining; specifically, the concept of an edge-computing-based production supervision system for the aerospace industry using a tool and cutting process condition monitoring system. The new concept was developed based on experience gained during the implementation of research projects in Poland’s Aviation Valley at aerospace plants such as Pratt & Whitney and Lockheed Martin. Commercial tool condition monitoring (TCM) and production monitoring systems do not effectively meet the requirements and specificity of the aerospace industry. The main objective of the system is real-time diagnostics and sharing of data, knowledge, and system configurations among technologists, line bosses, machine tool operators, and quality control. The concept presented in this paper is a special tool condition monitoring system comprising a three-stage (natural wear, accelerated wear, and catastrophic tool failure) set of diagnostic algorithms designed for short-run machining and aimed at protecting the workpiece from damage by a damaged or worn tool.

## 1. Introduction

Rapid digitization is changing societies around the world, including how we communicate, what we buy, how we live, and how the production system works. For the first time in history, digital technologies have connected more and more industries with numerous societies, and therefore are influencing the volume and quality of production as well as the functionality of products. Industry 4.0 has resulting in leading IT solutions in all aspects of production, enabling the ordering of specific products and entire related value chains. 

This involves the need to link production and logistics including storage, service, energy supply, linking networks of suppliers, etc. [1]. 

An important element of Industry 4.0 is cyber-physical systems (CPS) that use the Industrial Internet of Things to integrate new production organizational methods which results increased product personalization and production processes that require less participation of operators. An example of the architecture for implementing a CPS is presented in [2,3].

A practical example of a CPS is replacing the manual delivery of components in a production line with automated guided vehicles that transport components to the appropriate production cells. The use of RFID tags on manufactured products enables machine tools to automatically select a set of tools for a particular product, achieving full personalization of production and reducing the minimum production lot size to one piece. Another example is the use of the Industrial Internet of Things (IIoT) technology to implement a wireless sensor network in a plant that can monitor production, detect anomalies, such as vibrations, or even energy consumption by machinery and technological installations [3,4,5]. By obtaining data from a CPS and using appropriate analytical software, it is possible to optimize production within a factory, as well as to create performance benchmarks for all production units of companies throughout the world. 

With the development of new technologies, it is becoming increasingly common to support or replace traditional methods for supervising production processes with computerized and automated methods. Therefore, an important element of an Industry 4.0 system in a manufacturing factory is the production line supervision system which minimizes defects, increases the repeatability, optimizes machining conditions, and reduces failure rates and downtime of machine tools, resulting in increased machining efficiency, while reducing costs. In turn, supporting supervision of a production process improves utilization of the machinery park (including planning of repairs), achieves better control of the production process, and minimizes the time required for documenting the process. 

There are many commercial systems that perform TCM tasks [6,7,8,9,10] including:

- Cutting edge wear diagnostics (end of life detection);
- Detection of catastrophic tool failure (CTF);
- Diagnostics of the shapes of metal chips;
- Detection of excessive vibration;
- Others (built-up edge and collision detection).

Tool condition monitoring (TCM) systems have been designed as commercial systems by manufacturers such as Montronix, Nordman, Artis, and Digital Way [7,8,9,10].

Manufacturers offering commercial diagnostic systems, or their components, have solved the problem of measurement sensors quite well. Sensors are available for cutting forces and related quantities such as torque or power consumed by drives, acoustic emission, and vibration, which have measurement ranges suitable for the cutting process and are immune to conditions in the cutting zone [7,8,9] table among the sensors is an easy-to-install machine tool drive power sensor from Digital Way [10] with higher accuracy and wider bandwidth than Hall effect-based sensors offered by other manufacturers. There is an extensive range of hardware for tool condition monitoring, such as data acquisition cards, data processing units, as well as HMI (human–machine interface) panels, for service and system panel management. Diagnostic systems are available in compact form, in which all the components are integrated, and as modular systems for the configuration of a whole set of systems installed on many machine tools on the shop floor [9,10]. Hardware components are still being developed, but they are no longer a barrier to wider industrial use of TCM systems. This barrier is diagnostic algorithms involving signal processing and data analysis through to diagnosis, which remain very simple, based on a single signal feature (e.g., the mean value) and practically unchanged in commercial systems since the late 1990s. In addition, setting system operating parameters is usually quite cumbersome and requires extensive operator experience [6]. In commercial, real-world solutions, the user must match the diagnostic parameters to the specific machining task, which requires the user to have an appropriate level of knowledge in this area and to take a significant amount of time at the start of each new production to adjust the diagnostic system. Such strategies require precise trial-and-error boundary determination, and can be effective for high-volume, stable processing of easily machinable materials, such as in the case of Digital Way systems used at the FCA Poland SA automotive factory in Tychy. For CTF detection in commercial systems, strategies are used that are based on different types of boundaries. The floating boundary strategy is one of the most effective strategies which has been adopted in Nordmann’s systems; unfortunately, it is only suitable for machining where many operations on the tool life are performed. In the Nordmann example there are more than 500 operations [8]. In general, their reliability is regarded as unsatisfactory [11].

Studies have been conducted that used very sophisticated methods for tool condition monitoring [12,13,14,15] and process optimization [16]. Advanced, modern signal analysis methods are used to determine signal characteristics. Similar to commercial systems, analog electrical signals are preprocessed (amplification and anti-aliasing filtering) and converted into a digital representation, i.e., time series. Several signal features can be extracted from these time series in time, time-frequency, or frequency domain. In the time domain, statistical signal features are most often used such as average, effective value (root mean square (RMS)), power, amplitude, crest factor, variance, skew, and kurtosis [17,18,19,20], In addition, analysis of variance (ANOVA) [21], auto regressions (AR), moving average (MA), and auto regressive moving average (ARMA) models are used for signal features extraction in the time domain (e.g., [17,22]). In the frequency and time-frequency domains, signal features are usually extracted using a discreet or windowed Fourier transform (FFT and STFT) [17,23,24], discreet wavelet transform (DWT) [17,25,26,27], variational mode decomposition [14], or Hilbert–Huang transform (HHT) [25,28,29]. However, more and more sophisticated methods are continually being developed and proposed that supply more and more signal features, such as support vector regression [19,25], principal component analysis (PCA) [30,31,32], singular spectrum analysis (SSA) [33], permutation entropy [34], and fractal analysis [35,36].

Various methods of integration have been used for diagnostics based on determined features. The most common are the following artificial intelligence methods:

- Support vector machine (ν-SVM) for prediction of different tool wear states [37];
- Radial basis function-based kernel principal component analysis (KPCA_IRBF) and relevance vector machine (RVM) for a tool flank wear predictive model [38];
- An adaptive time window and deep bidirectional long short-term memory neural network for prediction of the tool failure zone in milling [14];
- A deep learning method based on multiscale feature fusion by parallel convolutional neural networks for tool wear prediction [39];
- An Ellipsoid ARTMAP (EAM) network model based on incremental learning algorithm for recognizing the wear states of milling tool [40];
- An HMM algorithm and multilayer perceptron (MLP) for accelerated wear [41];
- Recurrent neural networks for tool wear in turning [42].

A more extensive review of such methods can be found in [43].

There is a large discrepancy between strategies used in commercial systems based on single signals and laboratory strategies based on signal integration with results that are much more reliable. The main reason for this discrepancy is the low level of automation of laboratory algorithms. Developers of these algorithms perform many steps “manually” selecting, for example, signal segments for analysis, features, or neural network parameters for the analysis of a specific machining task. Although more efficient than before, the use of such an algorithm in a commercial system requires the operator to have considerable knowledge of diagnostic systems and there is a significant amount of work required to start each new production, which is not accepted by industry [11].

Another issue is that the TCM is a time-critical system. Especially, catastrophic tool failures need to be detected within milliseconds or faster. Therefore, not every type of supervision system can be connected directly to the cloud, and therefore sometimes edge computing is a better solution [44].

Today’s diagnostic systems are versatile and are used in a variety of industries that use machining, such as automotive, engineering, and shipbuilding. Each of these industries has its own specific production and the expectations from the diagnostic system are also slightly different. In this paper, we present the aerospace industry’s expectations of a machining process diagnostic system and the concept of a system that could meet these expectations.

## 2. The Needs of the Production Supervision System in the Concept of Industry 4.0 in the Aerospace Industry

The needs and specific requirements of the aircraft machining industry for a production supervision system have been identified during the implementation of research projects in Poland’s Aviation Valley, for example, at PZL Mielec (Lockheed Martin Corporation) and Pratt&Whitney Rzeszów [45,46]. In the aerospace metal cutting industry, the requirements for accuracy and quality are much higher than, for example, in the automotive industry, because the components are very expensive (approximately 150,000 euro per unit) and the failure of a component has catastrophic consequences (airplane crash). In the era of Industry 4.0, despite the obvious need for cutting process diagnostics systems in the aerospace industry, they are rarely used. The main reasons for this are several.

Due to the previously mentioned high component costs, the most important aspects are system reliability, accuracy of tool wear estimation, reliable CTF avoidance, and rapid detection even at the cost of many false alarms. Simple diagnostic strategies included in commercially available diagnostic systems are not considered to be effective enough by the aerospace industry; therefore, academic solutions are more effective, but they are still plagued by some problems.

An important aspect, as in all other industries, is the ease of use of diagnostic systems. Ideally, the system should be fully automatic. Commercial systems are not user friendly. Many algorithm settings including thresholds, signal segments to evaluate, and various parameters such as delays have to be manually selected by the operator. Machine tool operators have been known to stop using these systems precisely because of the complicated operation and, in this case, most academic solutions are based on custom solutions and not optimized for ease of use; often user interfaces are not developed, only signal processing software. These academic strategies are based on advanced signal processing methods, and often on artificial intelligence methods, which require the selection of many different parameters for their operation. These parameters are selected “manually” by researchers, which make the application of these strategies in a commercial solution even more complicated than current versions.

The aerospace industry works in short runs, even a few pieces in a single run. For this reason, the diagnostic system should be ready to operate without, or after learning on, one tool life cycle without the need to tune the system after subsequent tool life cycles, as is often the case in current commercial systems.

Another feature of aerospace machining is the large size of workpieces and difficult-to-machine materials. As a result, tool life is often only a few operations rather than hundreds as in the automotive industry. This, in turn, makes it necessary to replace the tool due to natural wear during the operation rather than afterwards. A diagnostic system should indicate the level of gradual tool wear more often than once per operation after completion of the operation, as is the case in commercial systems.

In the aerospace industry, there are a large number of components with complex shapes that are machined on five-axis machine tools and it is impossible to install a wired load cell on the table of such a machine. There are several sensor solutions that use radio signal transmission technology, but often a transmitter with a built-in battery is connected to the sensor via a cable (such as the Montronix accelerometer). There are rotary dynamometers from Kistler that can be mounted on a spindle, but they are costly, and several sensors are required for diagnostics. In an industrial setting, this is too expensive and cumbersome. Manufacturing companies still lack proposals for wireless sensors that are small in size and can be easily installed as close to the machining area as possible. In addition, installing diagnostic sensors requires interference with the kinematic structure of the machine tool, such as disassembling the turret of a lathe or dismantling the table of a milling machine. If these are new machines, this can void the service warranty. Therefore, owners of machine tools are skeptical about installing additional sensors. For this reason, new solutions based on power sensors for machine drives are being developed, which do not interfere with the construction of the machine tool and their installation only temporarily takes the machine tool out of production.

The aerospace industry requires complete production records for every part, which is not required in other industries. This must include when the part was made, by whom, and on what machine, as well as all measurements of the part. If a system is used to monitor the condition of a tool, then, so are the indications of that system. Therefore, in the aerospace industry, a tool condition monitoring system should be part of a production line flow monitoring system that can collect all this information.

## 3. The Concept of an Edge-Computing-Based Production Supervision System in Industry 4.0

**Figure 1:**
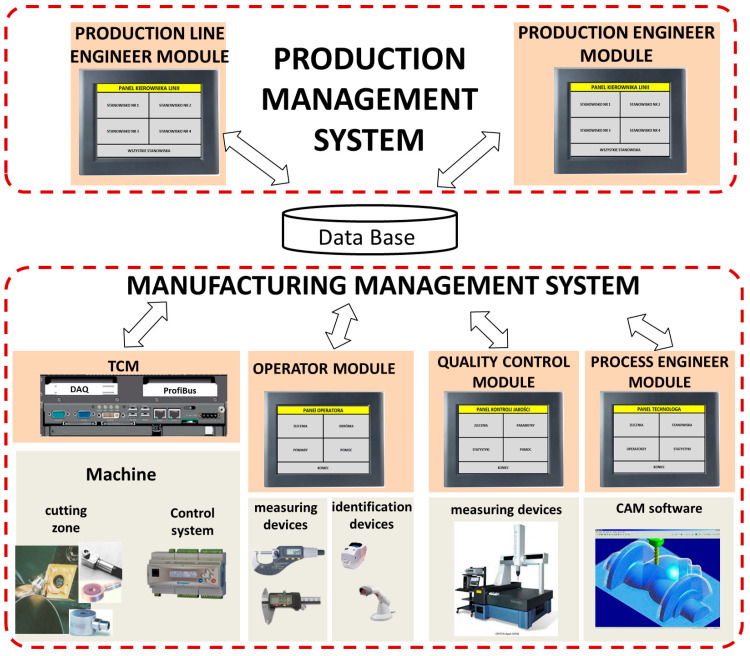
Diagram of the TCM concept [46].

Ensuring proper production supervision is particularly needed in the aerospace industry, where full documentation of the manufacturing process for each part is required, including information about who, when, and under what conditions it was manufactured or measured. The proposed concept for such a system is shown in Figure 1. It is divided into two main parts, i.e., the production management system and the production process supervision system. These systems exchange information with each other through a database. The production management system includes managerial positions, such as line manager or production manager. Using special applications with access to the knowledge base, management staff can, on the one hand, distribute orders for execution, provide technical and technological documentation, etc., and, on the other hand, collect information about the flow of products between stations, cells, machine loads, failures, etc.

The primary function of the tool condition monitoring and cutting process module is to predict the end of life and to detect catastrophic tool failure (CTF). 

However, there are autonomous systems that do not cooperate with master systems. A TCM system which enabled such capabilities was built at the Department of Automation and Metal Cutting at Warsaw University of Technology [15,45,46,47]. In addition to the basic functions mentioned above, it gathers information about who and when individual items were performed, the operational parameters, and the condition of the cutting edge for each operation. This enables a much more complete use of the TCM. By combining the information about the condition of the cutting edge with the results of measurements of manufactured items one can, for example, determine the impact of tool wear on this dimension. After the production of a particular batch, this mechanism can eliminate errors in training the system, normally performed by an operator. It is also possible to select workpieces to be measured, for example, in the case of excessive tool wear. On the basis of the shape of the diagnostic signals, the TCM system can identify operations that require correction (e.g., a sudden increase in force or vibration). In addition, by documenting operations in the form of digital data, it is possible to detect human errors, such as not replacing the tool at the right time and unplanned change of machining parameters through unauthorized change of control knob settings.

The operator module allows the operator to fully document the production process of a given component (serial number), starting from acceptance of the order, indication of the individual parts to be measured by quality control, recording of the measurement results, commenting on the individual parts, and finally closing the order. In addition, the module provides a list of serial numbers for a given order along with comments, parameters to be measured, nominal values, and acceptable deviations. The module also performs a measurement analysis, i.e., calculation of SPC parameters. The module uses scanners (barcode or QR) to read confirmation numbers and electronic measuring devices that automatically register measurement results, and enables communication between the operator and the TCM system. The operator sends information to the TCM system about cutting edge replacement, and receives information from the TCM system about tool wear status, occurrence of catastrophic tool failure or collision, and sensor status information.

The quality control module is used to document measurement results for parameters selected for inspection. It can read the confirmation number and measurement results directly from the electronic measuring instrument tools and scanners. The user of this module can designate a part as defective, quality control accepts measurement orders through this module, and the measurement results are stored in a database.

The process engineer module is used for ongoing control of processing results. One such module, running in the technologist’s office, interacts with several operator and quality control module systems installed at different production or inspection stations. The process engineer has access to measurement results from all cells, and therefore can control the stability of the process as well as provide detailed instructions on measurements or processing. Setting TCM parameters is also an important task of a process engineer.

**Figure 2:**
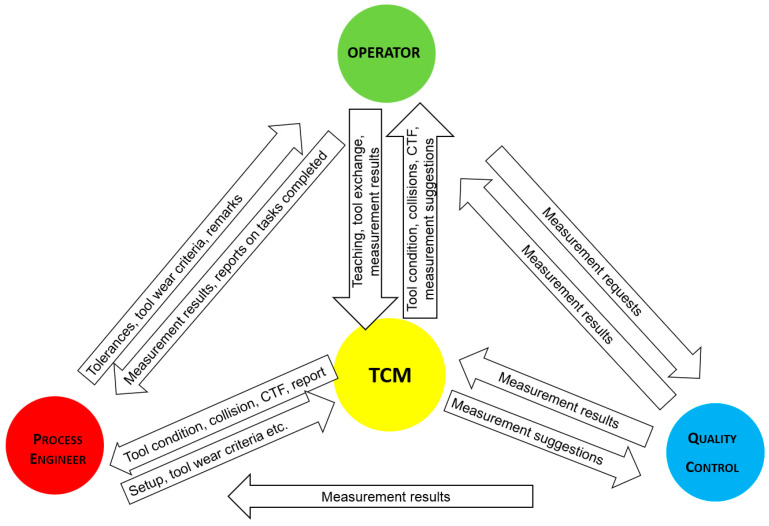
Information flow in the manufacturing process management system [46].

A production supervision system cannot function without appropriate data flow between the following modules: TCM, operator module, quality control module, and process engineer module. This is realized by means of a database, Figure 2. The following dependencies can be distinguished:

- The process engineer sends information about the diagnostic settings (e.g., sensitivity, selection of tools and sensors to be monitored, machining method declaration, tool definitions, etc.) to the TCM system, and also assigns individual tools to the machined surfaces on the part. On the basis of this information, the TCM system suggests validation of individual surfaces in the case of high cutting edge wear, defines the machining program according to which part is machined, and makes it available to the TCM system.
- The process engineer provides the operator with technological settings (guidelines for performing measurements, permissible deviations of measured parameters, tool wear criteria, and remarks).

- The TCM system transmits to the process engineer diagnostic signals, work progress reports, and results of machining process supervision on a given cell (e.g., date/time of execution of individual operations, operator’s name, tool wear values after execution of individual operations, and information on occurrence of emergency situations (collisions and catastrophic tool failures).
- The TCM system sends, to the operator, information about estimated tool wear, occurrence or threat of emergency situations (collisions and catastrophic tool damage), proposals for tool replacement, and estimated number of operations that can be performed at a given tool edge.
- The TCM operator provides information on tool replacement, and a suitably qualified operator also has the authority to teach the supervision system.
- The operator provides the process engineer with reports on completed tasks (orders and operations), measurement results, and comments.
- Quality control sends the measurement results to the process engineer.
- The TCM system can send suggestions to quality control about measurements at a site, such as when there is high tool wear.

## 4. Edge-Computing-Based Tool Condition and Cutting Process Module

**Figure 3:**
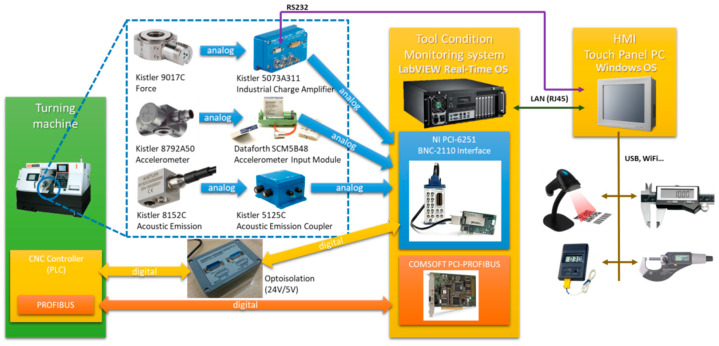
TCM system created by Warsaw University of Technology [46].

As mentioned above, the basic element of the manufacturing process control process is the supervision of the manufacturing process, for example, the cutting process, including the cutting tool. The advantage of a TCM system, which is part of the presented concept, is not only its cooperation with the superior system, but also its autonomy and efficiency in tool condition diagnostics (Figure 3). 

The elements that determine the effectiveness of a TCM system to the greatest extent are the diagnostic algorithms included in it.

**Figure 4:**
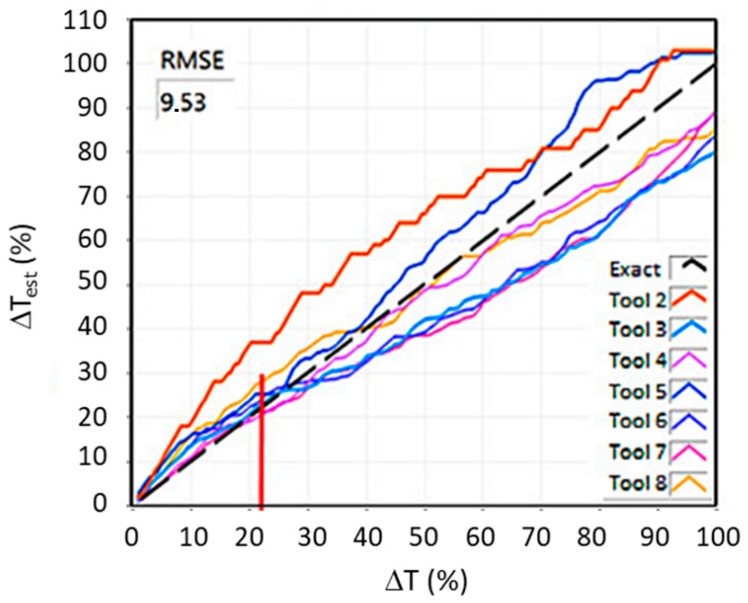
Results of the gradual tool wear estimation algorithm (GTW) [47].

**Figure 5:**
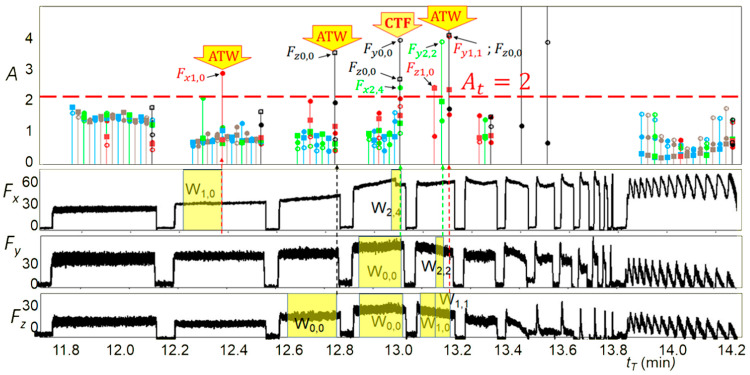
Results of the accelerated tool wear detection algorithm (ATW) [49].

**Figure 6:**
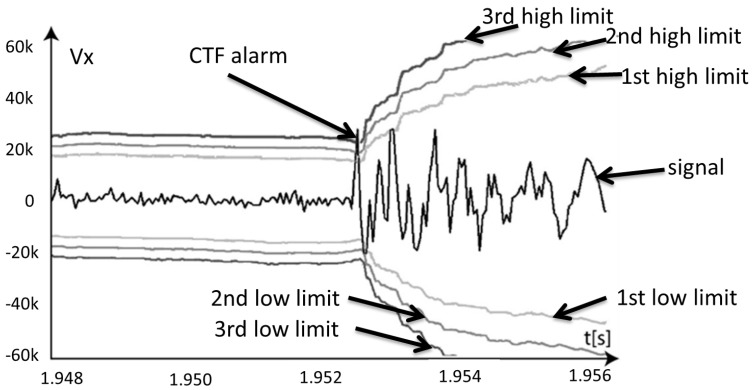
Performance results of the catastrophic tool failure detection algorithm (CTF) [50].

The concept of the system presented in this paper includes a three-level system of protection against working with a blunt tool as follows:

- **Estimation of gradual tool wear (GTW).** With a small number of operations in the life cycle, tool replacement may be necessary during operation. Thus, the algorithm must make a diagnosis about the state of the tool much more frequently than once per operation. An example of an algorithm that could be used here is the algorithm presented in [47]. Here, the tool wear diagnosis is presented up to 20 times per operation. Figure 4 shows the estimated tool wear using this algorithm for seven tools (from Tool 2 to Tool 8) and an ideal run (Exact). The tool wear rate presented by the system is used, here, as the used tool life ratio ΔT, which is an indicator that has long been used in tool condition diagnostics [48], shown in the figure as ΔT_est_ versus actual ΔT. Currently, the more popular RUL (remaining useful life) ratio is derived from the ΔT ratio according to the formula RUL = 100% − ΔT. The estimation error by the ΔT algorithm is shown as the root mean square error (RMSE). The number of operations per tool life is from seven to ten, and the tool wear estimates are much higher. The solution performs well from about five operations per tool life. Further development of diagnostic algorithms should lead to even lower numbers of operations per tool life, even below one, because such cases also occur in the aerospace industry.
- **Detection of accelerated tool wear.** Accelerated tool wear (ATW) often precedes the occurrence of catastrophic tool failure (CTF) and detecting it could prevent CTF. The ATW phenomenon is much more dynamic than GTW, therefore, the diagnosis is made much more frequently, at most within a few seconds and the faster the better, for example, the algorithm proposed in [49] can be used here. This algorithm detects accelerated natural wear and very small chipping (ATW) as well as larger chipping and tool breakage (CTF). The diagnosis is made every second. The results of this algorithm are shown in Figure 5. It shows the waveform of the signal shape change rate A in windows of different length Wi, j, where i is the number of passes and j is the number of windows in a pass. It also shows the courses of cutting forces Fx, Fy, and Fz on which diagnostics were performed. In addition, it indicates whether exceeding the threshold A = 2 triggering the alarm was caused by ATW or CTF. This algorithm can work even if there is less than one operation per tool life, but several operations must be used for learning anyway. Further development of this type of algorithm should lead to algorithms that do not require learning.

- **Detection of catastrophic tool failure.** The CTF must be detected in a fraction of a second, otherwise the workpiece may be damaged. The shorter the response time of the system, the lower the probability of this failure. For example, the algorithm developed in [50] can be used here. This algorithm can work based on force and/or vibration sensor signals. The latter is only effective with stable processing. Figure 6 shows how an algorithm like this works with the example of a vibration sensor signal. The waveform of the signal, the dynamic limits determined from the current dynamic component, and the additional coefficient obtained by the learning process are shown. The training can be performed on life periods of arbitrarily small number of operations. The CTF alarm is reported, here, less than 0.04 s after its occurrence. Further development of these algorithms should focus on eliminating the need to learn the system while being resistant to signal disturbances caused by changes in the cross-section of the machined layer or other reasons.

With this approach there is a high probability of avoiding machining with a defective tool, which is very important for the aerospace industry.

The TCM, presented in this study, uses fully automatic tool condition diagnostics based on multiple signal features for different machining methods and for different forms of cutting-edge wear. Proper communication with the machine tool is required to fully automate the TCM. The proposed set of algorithms for the presented TCM gives the possibility of automatic production completely without, or with limited, participation of an operator.

The tool wear detection algorithms used in the presented TCM system operate fully automatically, without unnecessary involvement by operators and technologists, and even without their knowledge. The developed TCM only requires the operator to mark tool changes. The basis of the algorithms consists of the following steps:

- Detection of cutting, i.e., the system automatically recognizes cutting and separates cutting periods into signals from fast feeds, as well as run-up and run-down;
- Automatic selection of signal segments with a fixed waveform [51];
- Determination of hundreds of signal features from the selected signal segments;
- Automatic selection of useful features (correlated with the tool condition) and elimination similar features (correlated with each other) for each individual segment [52];
- Determination of the used part of tool life for each feature separately and integration of indications into one evaluation, displayed to an operator as a percentage of tool wear.

Detection of catastrophic tool failure is a separate algorithm that operates in real time. The system detects such a failure within milliseconds and sends an emergency feed stop signal and a tool retract signal to the machine control system.

For proper and autonomous operation of the system, it is necessary to ensure that this system can communicate with the machine control system to provide the system with current information about machine operating parameters as follows:

- The name of the machining program;
- Two-state information on the status of operations, i.e., the start and end of the machining program and the start-stop of the working feed with division into individual axes;
- Information about the values of feed rates in individual axes, rotational and cutting speed, the number of the currently executed block, identification of the currently working tool (at least the number of the tool socket, and preferably the full name of the machining tool);
- The control system should have eight digital line outputs that transmit information about special functions from the level of the program executed by the machine tool;
- The control system should have two digital inputs, i.e., one to stop the feed rate and retract from the contour of the workpiece, and one to operate a special function in the machine tool program to wait for a “high” signal to appear at the digital input.

It is desirable to be able to save the machining program in parametric form so that parameter values (e.g., feed rate) can be corrected by the diagnostic system.

The diagnostic system should include a real-time data acquisition and processing module, a data visualization module, and a communication module with the machine tool and access to technological data. The diagnostic results should be available at least locally, however, it is strongly emphasized that, according to the Industry 4.0 paradigm, this is inefficient and the results should be exported to a database or cloud application.

## 5. Summary

The concept of a production supervision system based on edge-computing technology, as presented in this paper, and using a three-level algorithm of the tool estimating GTW and detecting ATW and CTF, would avoid production shortages and increase productivity while maintaining the required production quality. 

It is tailored to the requirements of the aviation industry, with a focus on minimizing defects in machining of expensive parts made with hard-to-machine materials and high precision requirements, as well as to the specificity of aviation production in which there are small series production and limited operations per one tool life. This system speeds up and minimizes the risk of error in making accurate production documentation and quality measurements of each manufactured part, which is required in the aerospace industry. Full documentation and supervision of the process parameters allows for its certification and a significant reduction in the number of measurements of parts, which contributes to improving the economics of production. The requirements presented in this paper, the specificity of the aerospace industry, and the suggested directions for developing diagnostic systems are the starting point for conducting work on new diagnostic systems in research centers.

## Data Availability

Not applicable.
